# Hot spots and frontiers in bone-tendon interface research: a bibliometric analysis and visualization from 2000 to 2023

**DOI:** 10.3389/fsurg.2023.1326564

**Published:** 2024-01-24

**Authors:** Hao Xiao, Boyuan Wen, Dong Yan, Quansi Li, Yujie Yang, Xianye Yin, Deyu Chen, Jiachen Liu

**Affiliations:** ^1^ XiangYa School of Medicine, Central South University, Changsha, China; ^2^ School of Journalism and Communication, Hunan University, Changsha, China; ^3^ Department of Orthopedics, Xiangya Hospital, Central South University, Changsha, Hunan, China; ^4^ Center of System Biology and Data Information, School of Basic Medical Science, Central South University, Changsha, Hunan, China

**Keywords:** bone-tendon interface, enthesis, trends, visualization, bibliometrics

## Abstract

**Objective:**

In this research, we investigated the current status, hotspots, frontiers, and trends of research in the field of bone-tendon interface (BTI) from 2000 to 2023, based on bibliometrics and visualization and analysis in CiteSpace, VOSviewer, and a bibliometric package in R software.

**Methods:**

We collected and organized the papers in the Web of Science core collection (WoSCC) for the past 23 years (2000–2023), and extracted and analyzed the papers related to BTI. The extracted papers were bibliometrically analyzed using CiteSpace for overall publication trends, authors, countries/regions, journals, keywords, research hotspots, and frontiers.

**Results:**

A total of 1,995 papers met the inclusion criteria. The number of papers published and the number of citations in the field of BTI have continued to grow steadily over the past 23 years. In terms of research contribution, the United States leads in terms of the number and quality of publications, number of citations, and collaborations with other countries, while the United Kingdom and the Netherlands lead in terms of the average number of citations. The University of Leeds publishes the largest number of papers, and among the institutions hosting the 100 most cited papers Hospital for Special Surgery takes the top spot. MCGONAGLE D has published the highest number of papers (73) in the last 10 years. The top three clusters include #0 “psoriatic arthritis”, #1 “rotator cuff repair”, and #2 “tissue engineering”. The structure and function of the BTI and its key mechanisms in the healing process are the key to research, while new therapies such as mechanical stimulation, platelet-rich plasma, mesenchymal stem cells, and biological scaffolds are hot topics and trends in research.

**Conclusion:**

Over the past 23 years, global research on the BTI has expanded in both breadth and depth. The focus of research has shifted from studies concentrating on the structure of the BTI and the disease itself to new therapies such as biomaterial-based alternative treatments, mechanical stimulation, platelet-rich plasma, etc.

## 1 Introduction

The bone-tendon interface (BTI, also known as enthesis) is the insertion point of tendons and ligaments in the bone and is an important structure for the transmission of mechanical forces from skeletal muscle to bone, and therefore a vulnerable part of the body (1). BTI is hierarchical and can be morphologically divided into four successive layers including tendons, uncalcified fibrocartilage, calcified fibrocartilage, and bone (2, 3). This complicated mechanical transfer system can regulate mechanical loads and decrease stress concentrations between bone and tendon (4). BTI is difficult to repair after injury, mainly attributed to (1) structural complexity, (2) lack of blood supply at the area of tissue recovery, and (3) slow and insufficient regeneration of the fibrocartilage layer. After injury, BTI proliferates through continuous pathological repair of fibers and blood vessels, forming a scar, that lacks the structural and functional properties of the original BTI and is prone to repeated injury (4). Rotator cuff tears (RCT) are a common reason for shoulder pain and dyskinesia (5). More than 17 million people in the U.S. have rotator cuff disease, which contributes to limitations in their ability to work and function in daily life (6). Rotator cuff reconstruction surgery is an essential therapy for rotator cuff disease (7). Currently, more than 450,000 rotator cuff reconstruction surgeries are performed annually in the United States, and this number continues to increase (8). About 30% of people over the age of 65 have RCT, and the probability of RCT increases with age (9). However, the re-tear rate after rotator cuff reconstruction is as high as 16%–94% (10, 11). The most significant cause of re-tearing is suboptimal healing of the BTI (12). Promoting the proper restoration and healing of the BTI is critical to treating RCTs and preventing re-tears after reconstruction. More than 120,000 anterior cruciate ligament (ACL) injuries are reported in the United States each year, primarily occurring during high school and university, which severely impacts adolescents’ daily lives (13). BTI is an influential factor in the postoperative outcome and prevention of recurrent ACL tears during ACL autograft reconstruction (12, 14). Impairment of BTI is an important pathologic feature of psoriatic arthritis, and pathological activation of the IL-23/IL-17 axis triggers an inflammatory response in BTI, and counter-regulation between the Th17 and Th2 pathways in the BTI region is involved in the development of psoriatic arthritis (15, 16).

Therefore, an increasing number of studies have addressed BTI and its related areas, and relevant literature continues to be published in academic journals. Some studies have explored tissue engineering and mechanical stimulation to promote BTI healing (17–21). However, with the increasing number of BTI research reports, searching quickly and efficiently for the current state of research in related fields has become a more realistic problem for researchers. Researchers also need a tool that fully utilizes the functions of retrospective summary and forward-looking prediction to deeply explore the characteristics of literature from the big data level. Researchers also need a tool that fully utilizes the functions of retrospective summary and forward-looking prediction to deeply explore the characteristics of literature from the big data level. Bibliometrics analysis and visualization provide an essential, feasible, and systematic method for determining the significance of published literature by displaying basic information about the papers, connections between authors, institutions, and countries, as well as current and future developments in related fields (22–28). Utilizing the results of bibliometric analysis not only helps researchers to be aware of global research trends and to have access to sources of research information but also helps researchers to understand the strengths and weaknesses of their research and to quickly capture research priorities, hotspots, and trends (29). At present, there is a deficiency in summarizing and evaluating the literature characteristics, research directions, research depth, and research hotspots of BTI research. Therefore, it is highly necessary to determine the concentration hotspots, research frontiers, and emerging trends in the field of BTI research in general, which can provide references for future research.

In this study, BTI-related papers in the Web of Science core collection (WoSCC) between 2000 and 2023 were screened and analyzed by using the R packages, VOSviewer, and CiteSpace, including the types of included papers, the overall trend of publications, countries/regions, institutions, authors, journals, keywords, and so on. The research progress of BTI was explored from the perspectives of bibliometrics and visualization analysis, aiming to help readers quickly gain relevant knowledge about BTI, and for readers who are new to the field or are not deeply involved in it, the article undoubtedly provides an effective and convenient way. This paper can help readers understand what the field of BTI has been studied before, what it is doing now, how it will be developed in the future, whether it is suitable for one's own research direction as well as access to high-quality articles and journals in the field, which is also beneficial for the submission of papers.

## 2 Materials and methods

### 2.1 Data sources and search strategies

The retrieved data for statistical analysis were selected from the Web of Science Core Collection (WoSCC), which provides citation searches, allows access to multiple databases referencing interdisciplinary research, and allows for in-depth exploration of specialized areas.

The search strategy was “Bone tendon interface (Topic) OR enthesis (Topic) AND 2000-01-01/2023-09-23 (Publication Date)”.

### 2.2 Inclusion and exclusion standards

**Figure 1:**
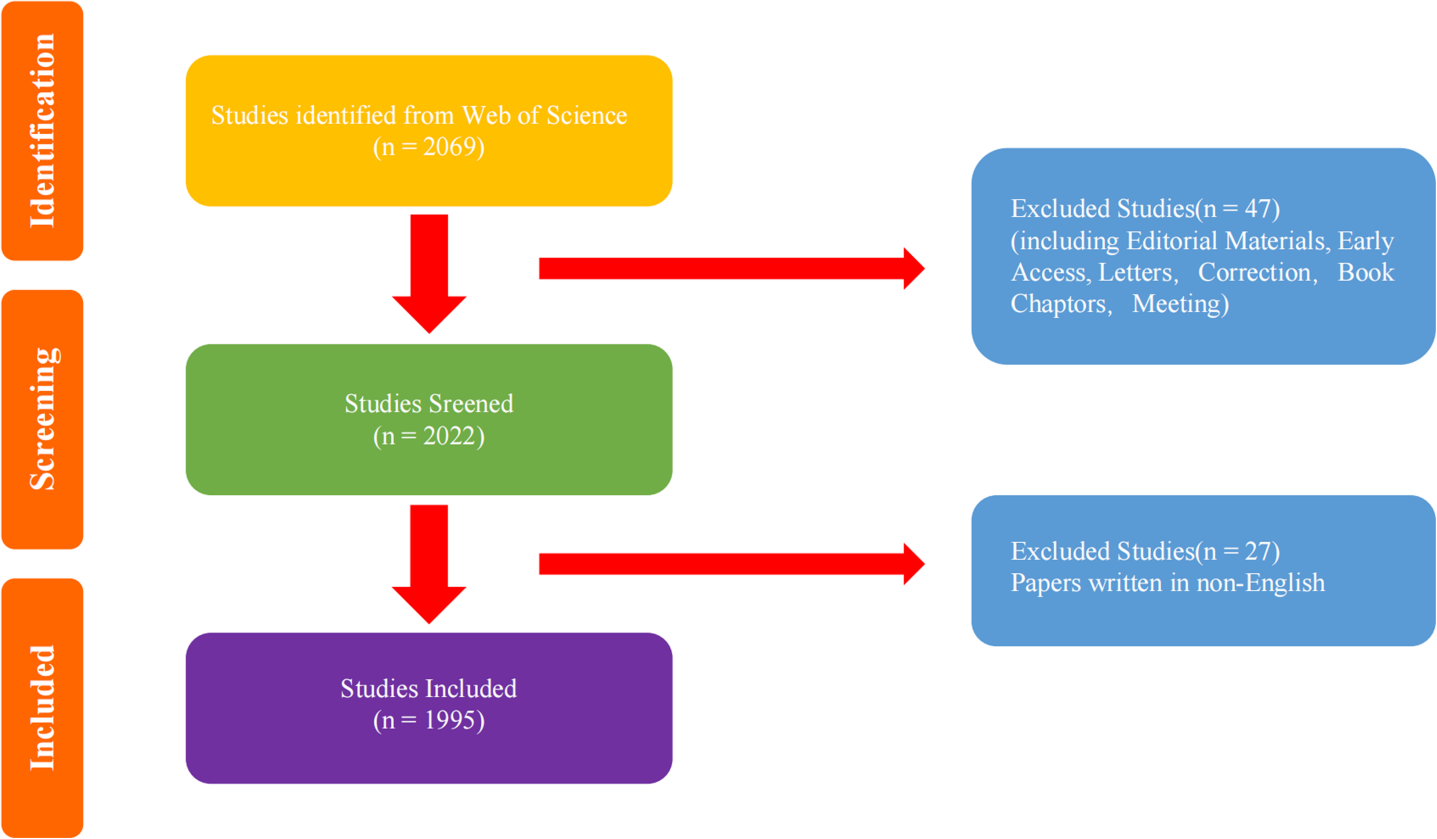
Literature screening process.

A total of 2,069 documents were initially searched, and 2,022 documents were filtered out by filtering less representative document types, such as Editorial Materials, Early Access, Letters, Correction, Book Chapters, and Meeting, which contains the following document types: Articles, Review Articles, and Meeting Abstracts. 1,995 documents were screened out as English-only documents. If the term “Bone tendon interface” or “enthesis” did not explicitly appear in the title, abstract, or index entry, it was not included in the 1,995 documents. Full-text records and cited references were selected in plain text format and downloaded for further analysis. Figure 1 shows the detailed screening process.

### 2.3 Data analysis

Bibliometric analysis and visualization were performed by using WoSCC, Citespace V6.2.4R, VOSviewer 1.6.19, WPS, and R Bibliometrix software packages.

From the analysis results of the R Bibliometrix package, we can easily acquire information about the number of papers published annually, which can then be exported to WPS to calculate the annual growth rate. In addition, data related to countries/regions, institutions, authors, journals, and keywords were also obtained from it.

#### 2.3.1 H-index obtained via WoSCC’s citation reports

Citespace is an interactive visualization tool that combines information visualization methods, bibliometrics, and data mining algorithms, which is a unique and influential application in the field of information visualization and analysis, providing clinicians and researchers with a more intuitive and scientific analysis of the field (30). We used Citespace for keyword clustering analysis and dual map overlay analysis and made the information more intuitive through timeline view or time zone view, and also obtained a table of keyword citation outbreaks. The settings are as follows: time span (2020–2023), Years per slice (1), term source (Keywords) and pruning (none); clustering labels are extracted by light semantic indexing and log-likelihood ratio algorithms, and other labels follow the default values (Strength: Cosine; Scope: Within Slices; g-index: k = 25; top *N* = 50; top N% = 10.0%; the maximum number of selected items per slice 100; Visualization: cluster view-static and show merged network). In Citespace, node size represents co-occurrence frequency. Nodes have multiple colors which represent different years, from 2000 to 2023, varying from gray to red over time.

VOSviewer is also a bibliometric software that can create visual maps and can be displayed in three ways: clustering type, overlay, and density color. In this bibliometric study, we used VOSviewer to draw visual images of countries/regions, institutions, and authors, where the size of the nodes indicates the frequency of co-occurrence and the color of the nodes indicates the clustering. Additionally, links reveal co-occurrence relationships, and the thickness of the links depends on the calculated intensity value, which is proportional to the association of the two nodes.

## 3 Results

### 3.1 Volume and trend of article publications

**Figure 2:**
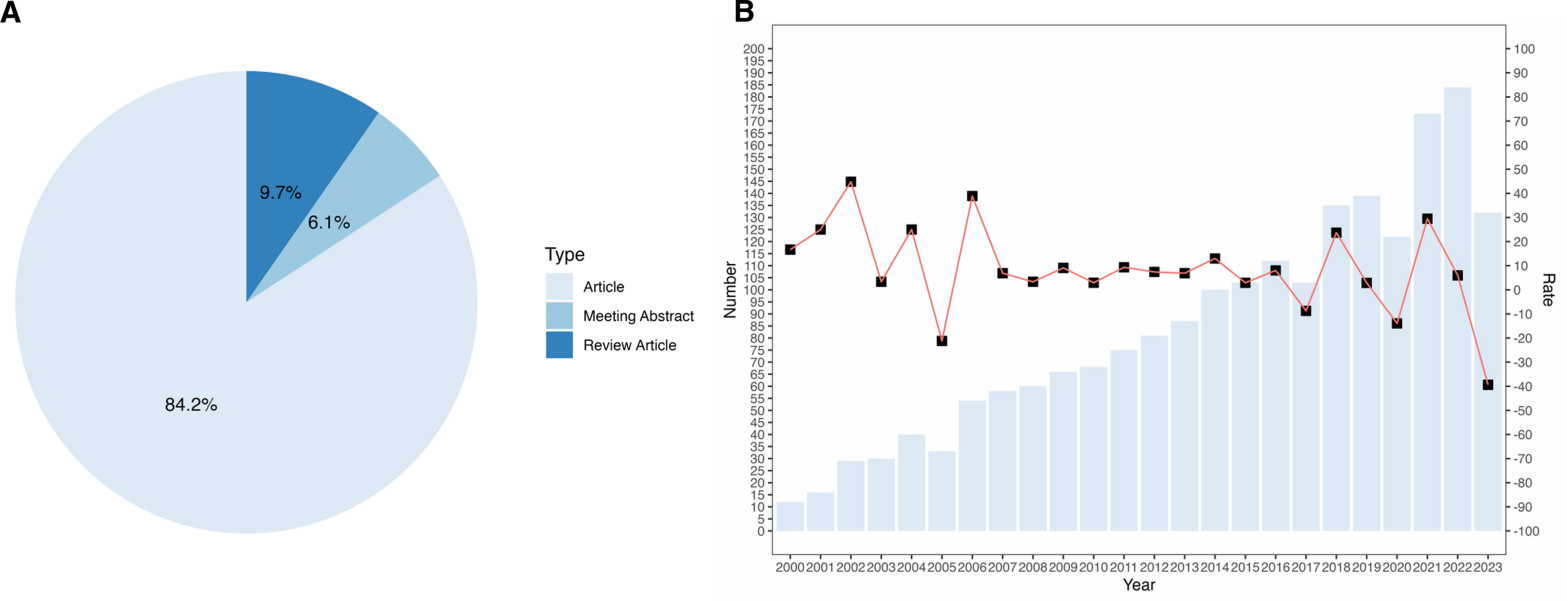
Types of literature and their issues and trends. (**A**) Types of literature included and their percentages. (**B**) Number of publications per year and their growth rate.

From 2000 to 2023, WoSCC has retrieved 1,995 BTI-related papers, of which 1,680 (84.2%) were “Article”, 194 (9.7%) were “Review Article”, and 121 (6.1%) were “Meeting Abstract” (Figure 2A). The number of papers published per year is an essential indicator of the rate of development of knowledge in the field and the trend of development in the field. The annual number of publications in the field of BTI is shown in Figure 2B. We can intuitively observe that there is a decrease in the number of published papers compared to the previous year in the three-time nodes, which are 2004–2005, 2016–2017, and 2019–2020. Since the screening literature ends on September 23, 2023, 2022–2023 is not counted as a decline, and judging from the growth rate in recent years, the number of publications in 2023 should maintain a continuous growth. Overall, the BTI field has maintained an overall trend of growth in the number of publications, with the highest growth rates from 2021 to 2022.

### 3.2 Number of citations to the 10 most influential papers

The total number of citations is an influential indicator of the number of times an article has been cited by other papers since its publication, which reflects the value of an article to some degree. Based on the literature citation analysis, Table 1 lists the top 10 highly cited papers. The number of citations for the top 10 cited papers ranges from 747 (“IL-23 induces spondyloarthropathy by acting on ROR-γt + CD3 + CD4-CD8-entheseal resident *T* cells”) to 285 (“Tendon to bone healing: differences in biomechanical, structural, and compositional properties due to a range of activity levels”). Interestingly, 5 of these 10 papers were reviews, and the remaining 5 concentrated on the clinical issues of BTI or enthesis. The most cited article (“IL-23 induces spondyloarthropathy by acting on ROR-γt + CD3 + CD4-CD8-entheseal resident *T* cells”), which was published in Nature Medicine in 2012, has an IF = 82.9, suggesting that IL-23 and the immune system have an important role in diseases caused by BTI abnormalities (31).

**Table 1:** Top 10 highly cited articles.

Rank	Article	Total citation	Publication year	Journal
1	IL-23 induces spondyloarthropathy by acting on ROR-γt + CD3 + CD4-CD8-entheseal resident *T* cells	747	2012	Nature Medicine
2	Tough bonding of hydrogels to diverse non-porous surfaces	679	2016	Nature Materials
3	Efficacy and safety of infliximab in patients with ankylosing spondylitis: results of a randomized, placebo-controlled trial (ASSERT)	644	2005	Arthritis and Rheumatism
4	Where tendons and ligaments meet bone: attachment sites (“entheses”) in relation to exercise and/or mechanical load	515	2006	Journal of Anatomy
5	Engineering complex tissues	428	2006	Tissue Engineering
6	The skeletal attachment of tendons–tendon “entheses”	380	2002	Comparative Biochemistry and Physiology. Part A, Molecular and Integrative Physiology
7	The anatomical basis for disease localisation in seronegative spondyloarthropathy at entheses and related sites	378	2001	Journal of Anatomy
8	Gradient biomaterials for soft-to-hard interface tissue engineering	291	2011	Acta Biomaterialia
9	Part I: footprint contact characteristics for a transosseous-equivalent rotator cuff repair technique compared with a double-row repair technique	288	2007	Journal of Shoulder and Elbow Surgery
10	Tendon to bone healing: differences in biomechanical, structural, and compositional properties due to a range of activity levels	285	2003	Journal of Biomechanical Engineering

### 3.3 Country/region, institution, author

**Figure 3:**
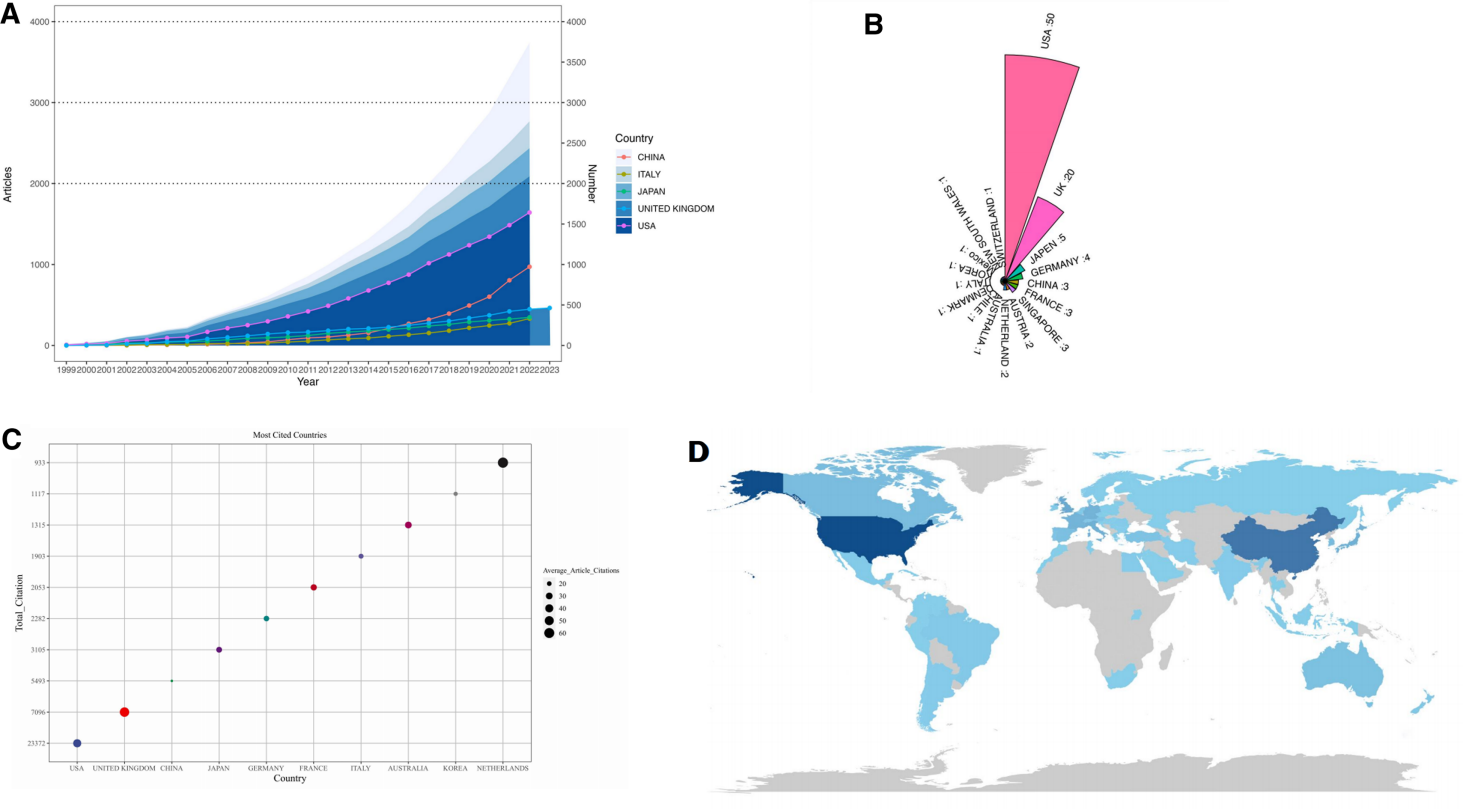
The status of publications in the field of BTI in countries around the world. (**A**) Line graphs and area graphs of the top 5 countries in terms of the number of articles issued per year. (**B**) The number of articles by country is included in the top 100 cited articles. (**C**) Bubble chart of the number of articles by country. (**D**) World map of the number of articles by country.

Visual information assists us in understanding the different teams and illuminates the collaboration between countries, institutions, and authors. Figure 3A shows the annual volume of publications and its trends for the five most productive countries/regions. The H-index is defined as an individual having published at least H articles with at least H citations each. Table 2 shows the top 10 countries/regions in terms of the number of publications published and their corresponding H-indexes. The top three countries in terms of the number of publications published are the USA, China, and the United Kingdom. Interestingly, China's H-index is not higher than the United Kingdom. The United States has the highest volume of publications and the number of publications has been increasing every year, China's number of publications was lower than that of Japan, the United Kingdom, and Italy until 2016, after which it surpassed all three countries. Japan, the UK, and Italy were roughly equal in terms of annual publications. Figure 3C shows the number of publications and average citations for the top 10 countries in terms of publications. Although the United States and China have the highest number of papers, their average citation is not as high as that of the United Kingdom and the Netherlands, indicating that these two countries are leaders in terms of the quality of their papers. Figure 3D presents the distribution of countries publishing BTI-related papers in the world, and the darker the color represents the higher the number of papers published. Generally speaking, North America, Europe, and East Asia are in the leading range in the world in terms of the number of papers published. When analyzing the countries from which the top 100 cited papers originate, the United States still takes the lead, followed by the United Kingdom, Japan, Germany, and China. Therefore, the United States has a large advantage in the field of BTI, and although China has several publications, the quality of its papers is not as good as that of countries such as the United Kingdom and Japan (Figure 3B).

**Table 2:** Top 10 countries/regions in terms of number of publications and H-index.

Rank	Countries/regions	Number of publications	H-index
1	USA	702	88
2	China	373	38
3	United Kingdom	193	50
4	Japan	150	37
5	Italy	140	35
6	Germany	126	30
7	France	113	31
8	Canada	79	23
9	Australia	73	28
10	South Korea	65	19

**Figure 4:**
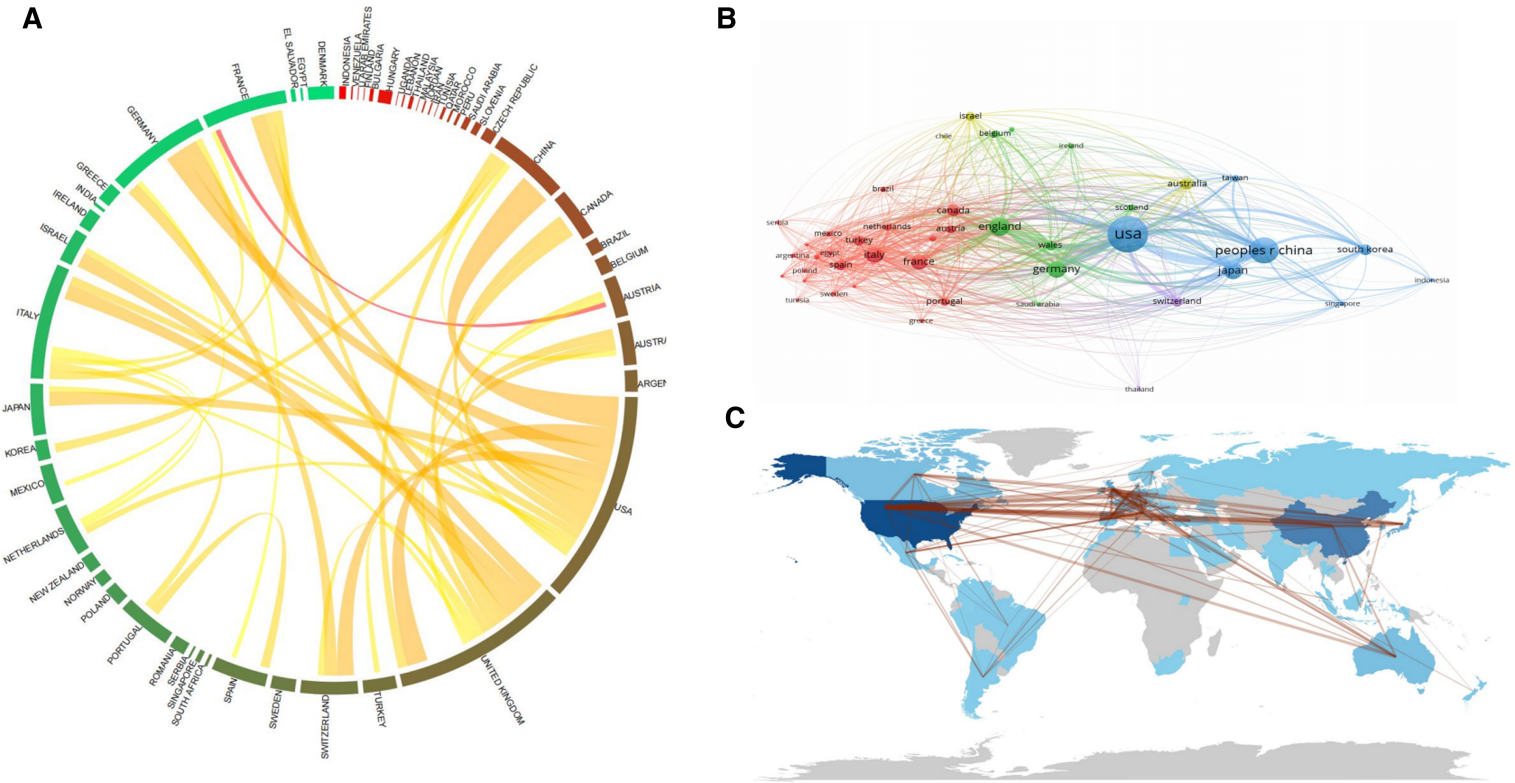
Cooperation chords between countries in the BTI field. (**A**) Chord diagram of cooperation among countries. (**B**) Relationship diagram of countries shown by VOSviewer. The same color indicates a close cooperation relationship. (**C**) World map of cooperation among countries. Connection indicates a cooperative relationship and more connections represent more collaborations. The color represents the number of published papers, and a deeper blue represents more published papers.

In the process of analysis, we found that close collaborative relationships between countries or regions are very common. Figure 4 describes the collaborative relationships between the countries which published BTI-related papers, indicating close cooperation between different countries and regions. The United States is the country with the most publications and also the country that collaborates most with other countries or regions, followed by the United Kingdom. Additionally, the United States collaborates particularly closely with China. Generally speaking, European countries collaborate more among themselves, the United States collaborates more with various countries in Asia, and Oceania and South America collaborate with all other continents.

**Figure 5:**
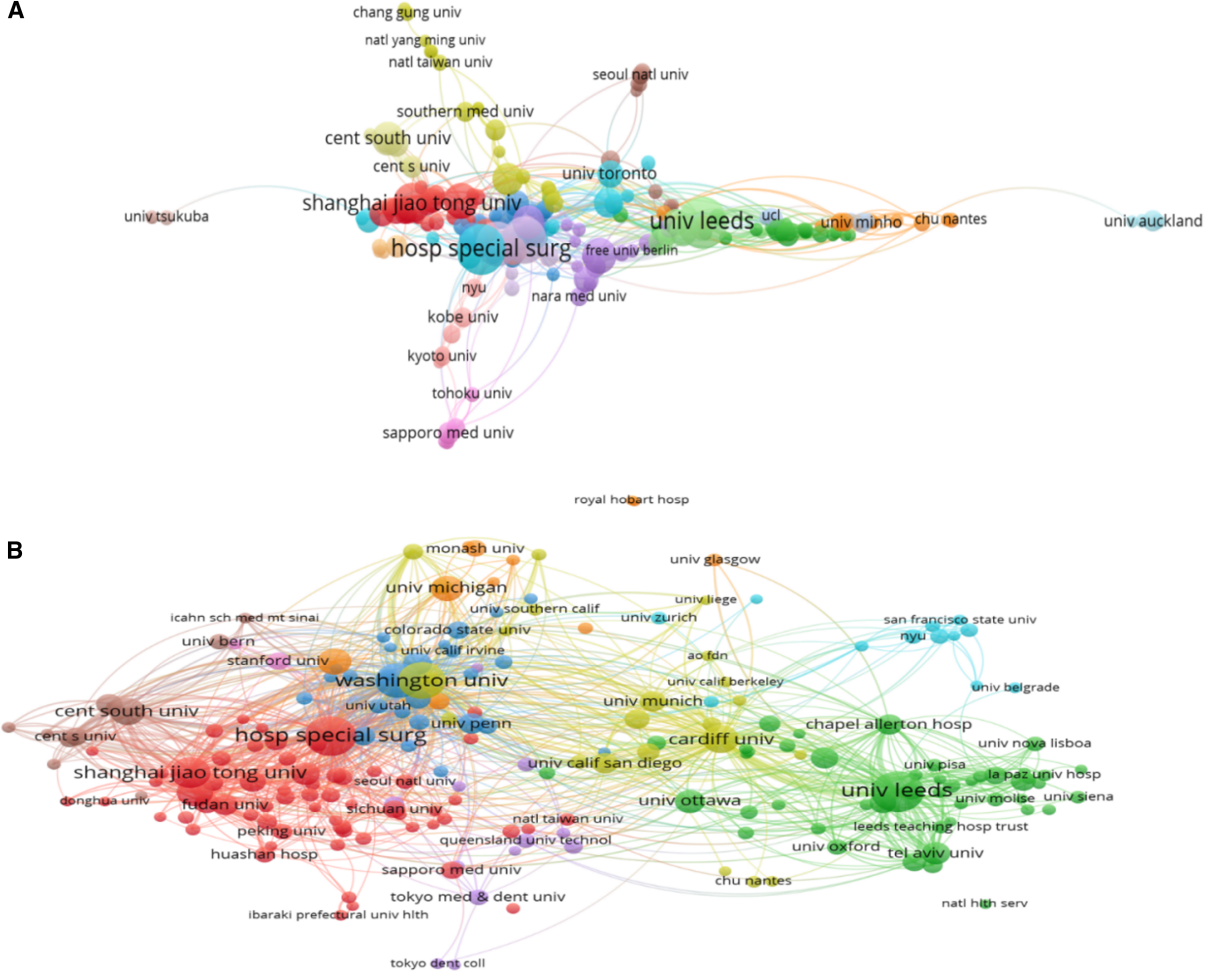
Collaboration graphs and co-citation relationships across institutions displayed by VOSviewer. (**A**) Collaboration graph across institutions. (**B**) Graph of co-citation relationships across institutions. Wires represent collaborative relationships, and the same color represents closer connections.

As Table 3 shows, the top 3 institutions that published the highest number of papers including the University of Leeds, the Hospital for Special Surgery, and University of California system, with 87, 74, and 67 documents, respectively. The top 10 institutions in terms of the number of papers published include four U.S. institutions, two Chinese institutions, and four U.K. institutions. When it comes to the H-index, the top three institutions are not the same as the institutions that published the highest number of papers, they are the Hospital for Special Surgery, University of Leeds, and Washington University. Although the United States had the most institutions in the top ten, no institution ranked in first place. Interestingly, when analyzing the institutions included in the 100 most cited papers, the institutional rankings changed dramatically. The top three institutions were Hospital for Special Surgery, Cardiff University, and Washington University (Table 4). Additionally, the top 10 also includes two UK institutions, six US institutions, one Dutch institution, and one Japanese institution. However, there are no Chinese institutions on the list. Collaboration between different institutions is recognized as a key factor in driving the successful development of large-scale research. At this point, there seems to be close cooperation between different institutions in different countries and regions. Over the past 2 decades, there has been close collaboration between institutions in China, such as Central South University, South Medicine University, and Shanghai Jiao Tong University. Institutions in Europe and institutions in North America have collaborated separately (Figure 5A). When analyzing the citation relationship of papers published by each institution, we found that Chinese institutions cited each other more frequently (red and brown nodes), UK institutions cited each other more frequently (green nodes), and US institutions cited each other more frequently (blue nodes) (Figure 5B).

**Table 3:** Top 10 institutions in terms of number of publications, the countries in which they are located and H-index.

Rank	Institution	Number of publications	Country	H-index
1	University of Leeds	87	United Kingdom	36
2	Hospital for Special Surgery	74	USA	37
3	University of California system	67	USA	34
4	Washington University (wustl)	61	USA	36
5	Columbia University	57	USA	31
6	Shanghai Jiao Tong University	55	China	18
7	Central South University	47	China	15
8	Cardiff University	45	United Kingdom	29
9	University Of London	37	United Kingdom	18
10	Chapel Allertion Hospital	36	United Kingdom	24

**Table 4:** Top 10 institutions that published the highest number of articles when analyzing the 100 most cited articles from 2000 to 2023.

Rank	Institution	Number of publications	Country
1	Hospital for Special Surgery	12	USA
2	Cardiff University	9	United Kingdom
3	Washington University	9	USA
4	University of Leeds	5	United Kingdom
5	University of Pittsburgh	5	USA
6	Columbia University	4	USA
7	Case Western Reserve University	2	USA
8	University Hospital Maastricht	2	Netherland
9	University of Pennsylvania	2	USA
10	Akita University School of Medicine	1	Japan

**Figure 6:**
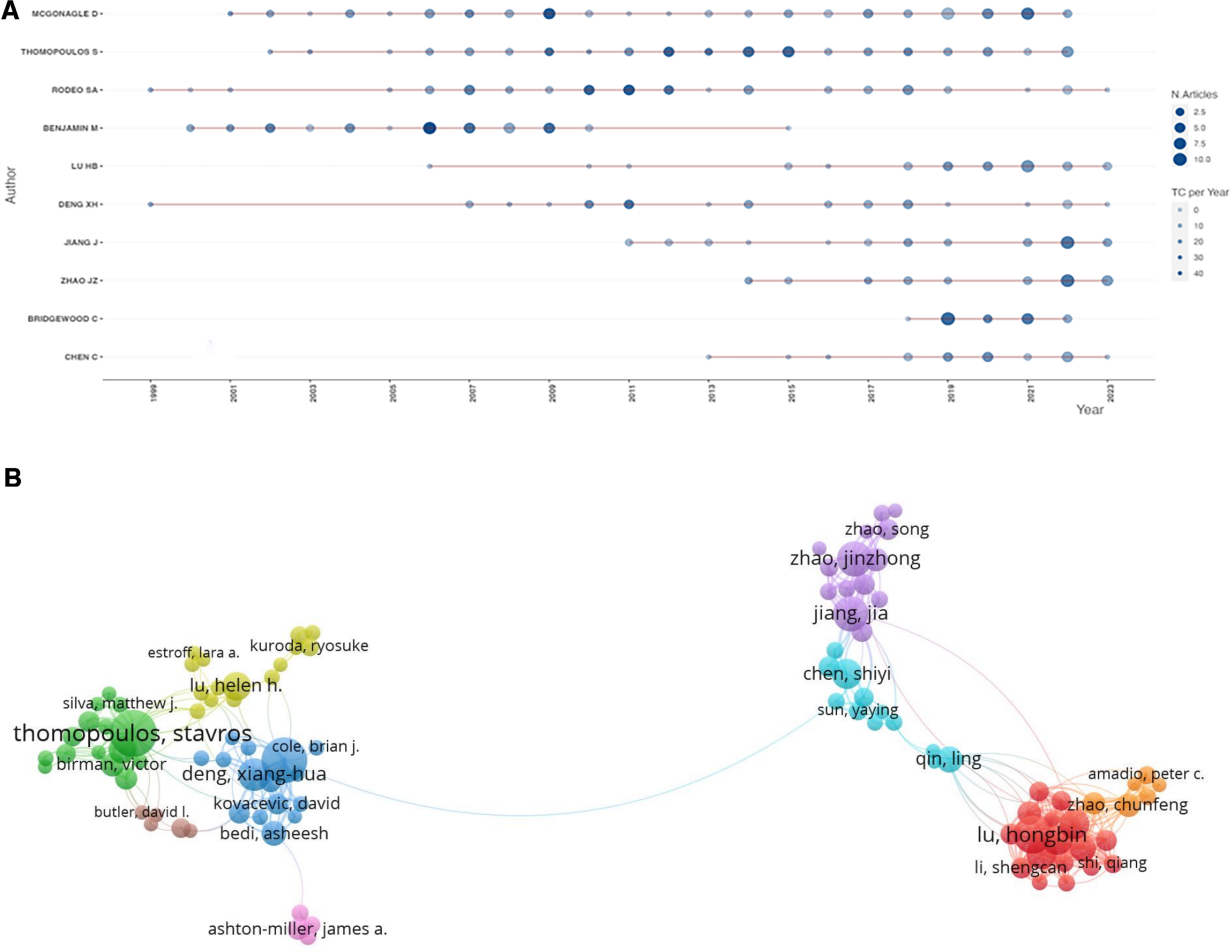
Authors’ posting volume and their partnerships. (**A**) Bubble chart of author posting volume. (**B**) VOSViewer displays the author partnership graph. Wires represent collaborative relationships, and the same color represents closer connections.

To identify the most influential experts in the field of BTI in the last 10 years, we ranked all authors according to their number of publications. The top 10 authors in the field of BTI in terms of publications are shown in Table 5 and Figure 6A. Among them, the top 3 authors are MCGONAGLE D, THOMOPOULOS S, and RODEO SA, who have published 73, 61, and 53 papers, respectively. THOMOPOULOS S own's the highest H-index. It is worth noting that the top 10 authors are all from the USA, UK, and China. The three authors from China published literature concentrated after 2015. When analyzing the authors included in the most cited papers in the field of BTI, THOMOPOULOS S, and MCGONAGLE D were still in the top 3. Still, their rankings were reversed, i.e., THOMOPOULOS S was ranked 2nd, MCGONAGLE D was ranked 3rd, and BENJAMIN M was the most numerous, with 7 papers (Table 6). The collaborative network analysis of authors is shown in Figure 6B. The authors’ collaborative network analysis categorized the authors into nine clusters, from which we identified several major research groups, notably THOMOPOULOS S et al, LU HB et al, ZHAO JZ et al, and DENG XH et al.

**Table 5:** Top 10 authors, number of publications, their country of origin and H-index.

Rank	Author	Number of Publications	Country	H-index
1	Mcgonagle D	73	United Kingdom	24
2	Thomopoulos S	61	USA	37
3	Rodeo SA	53	USA	33
4	Benjamin M	45	United Kingdom	21
5	Lu HB	33	China	12
6	Deng XH	31	USA	20
7	Jiang J	31	USA	11
8	Zhao JZ	30	China	14
9	Bridgewood C	25	United Kingdom	12
10	Chen C	24	China	8

**Table 6:** Top 10 prolific authors in the area of bone-tendon interface in analyzing 100 most cited papers from 2000 to 2023.

Rank	Author	Number of publications	Country
1	Benjamin M	7	UK
2	Thomopoulos S	5	USA
3	Mcgonagle D	4	UK
4	Rodeo SA	4	USA
5	Bedi A	2	USA
6	Gulotta LV	2	USA
7	Lu HH	2	USA
8	Park MC	2	USA
9	Tan AL	2	UK
10	Anderson K	1	USA

**Figure 7:**
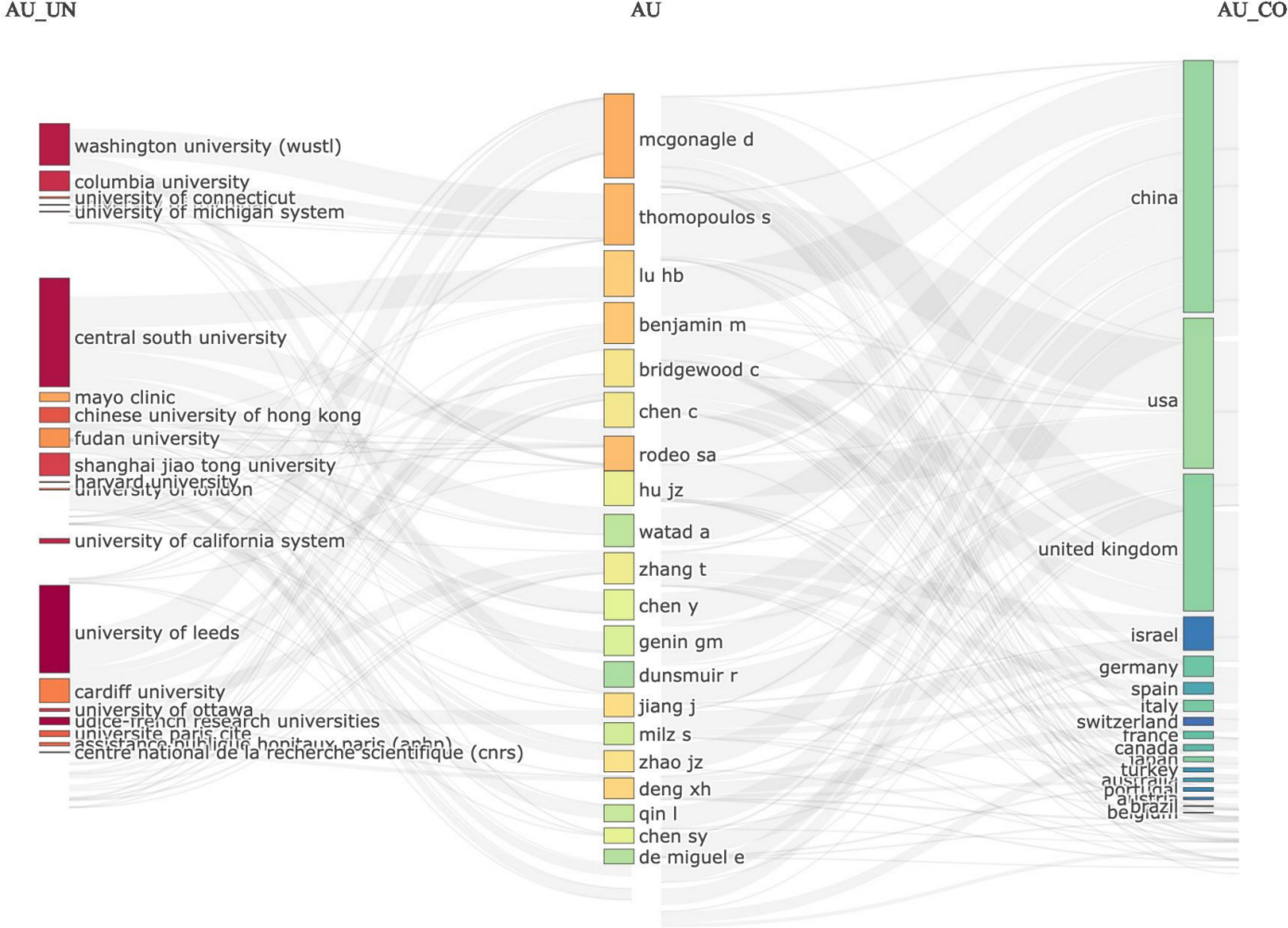
Sankey plot of countries, authors, institutions (threeFieldPlot).

Figure 7 shows the ThreeFieldPlot between country/region, author, and institution, which shows the relationship between the three more intuitively, such as Lu HB and Chen C, who are both from Central South University, MCGONAGLE D, who works mainly at institutions in the UK, and THOMOPOULOS S, who is located in the US.

### 3.4 Journals

**Figure 8:**
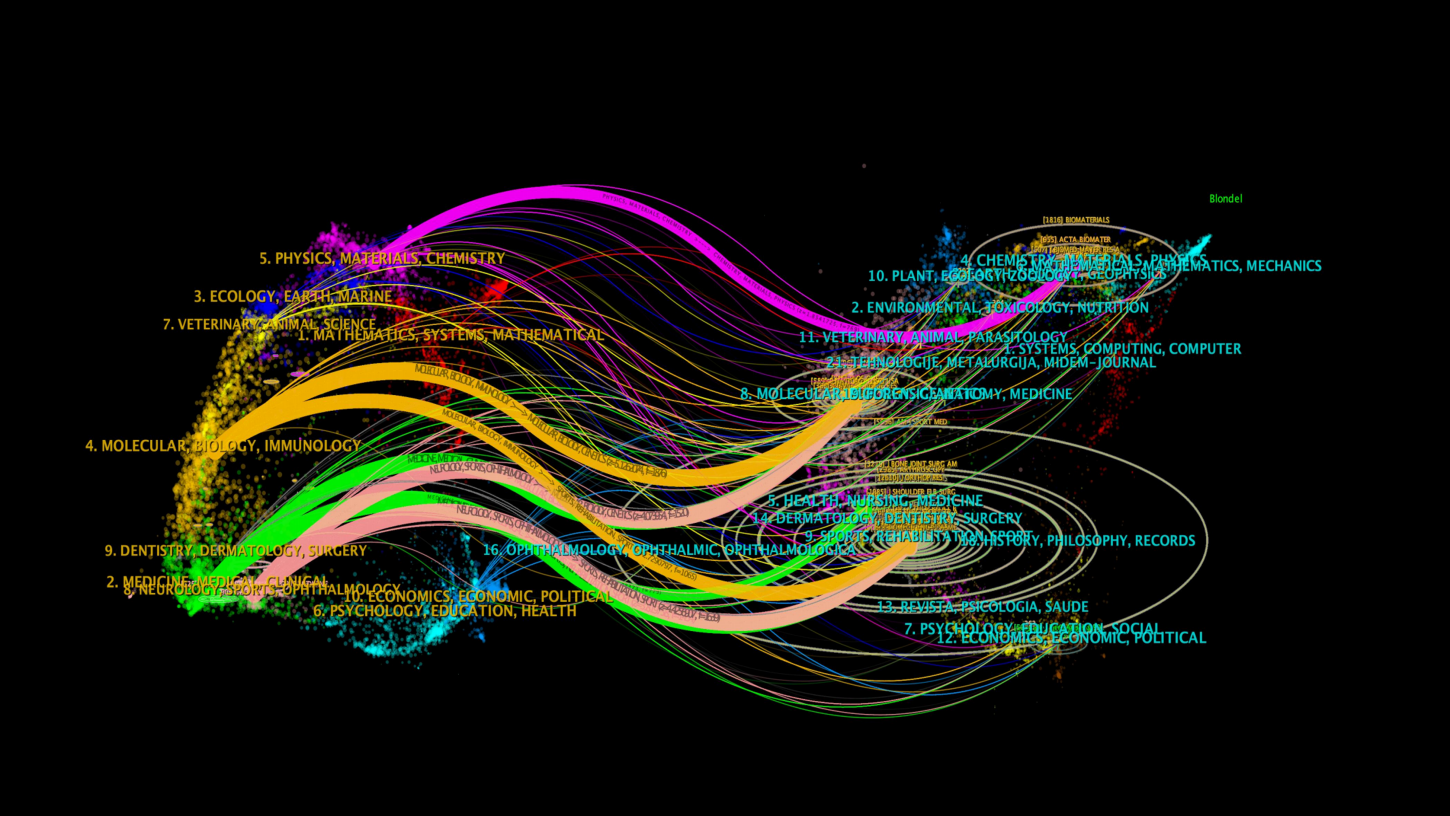
Dual-map overlay for the journal's domain. The size of the circle indicates the level of attention given to this field in BTI. The thickness of the line represents the number of references.

Table 7 lists the top 10 journals based on the number of citations. “The American Journal of Sports Medicine” was the most cited journal, followed by “Arthroscopy-The Journal of Arthroscopic and Related Surgery” and “Journal of Orthopaedic Research”, with 5,300, 3,134 and 2,501 citations, respectively. Additionally, the top 9 journals all had more than 1,000 citations. These journals’ 2023 impact factors ranged from 2.4 to 27.4, with almost all journals not exceeding an impact factor of 10, except for “Annals of Rheumatic Diseases” and “Biomaterials”. According to the JCR partitioning analysis, Q1 was 70% of the top journals in this ranking, Q2 was 10%, and Q3 was 20%. The journals are all related to bone, sports medicine, anatomy, and rheumatology. Figure 8 shows the DUAL MAP OVERLAY of the journals, as shown in the figure, the sizing citation literature is concentrated in the field of MEDICINE, MEDICAL, CLINICAL, the field of NEUROLOGY, SPORTS, and OPHTHALMOLOGY, the field of MOLECULOR BIOLOGY, IMMUNOLOGY and PHYSICS, MATERIALS, CHEMISTRY, while the cited literature is concentrated in the fields of SPORTS, REHABILITATION SPORT, MOLECULOR, BIOLOGY, IMMUNOLOGY and MEDICINE, MEDICAL, CLINICAL. MEDICAL, CLINICAL fields. We can see that the most important 4 paths, the SPORTS, REHABILITATION SPORT are often cited by the MEDICINE, MEDICAL, CLINICAL field and NEUROLOGY, SPORTS, OPHTHALMOLOGY field, the MEDICINE, MEDICAL. CLINICAL fields and PHYSICS, MATERIALS, CHEMISTRY fields are usually cited within the field.

**Table 7:** Top 10 journals in terms of publications and their impact factor, partitioning, frequency, total citations and average citations.

Rank	Journal	Impact factor	Journal citation reports	Frequency	Total citation	Average
1	The American Journal of Sports Medicine	4.8	Q1	119	5,300	44.5
2	Arthroscopy-The Journal of Arthroscopic and Related Surgery	4.7	Q1	70	3,134	44.8
3	Journal Of Orthopaedic Research	2.8	Q3	74	2,501	33.8
4	Annals of Rheumatic Diseases	27.4	Q1	58	2,209	38.1
5	Journal of Shoulder and Elbow Surgery	3.0	Q2	60	2,072	34.5
6	Journal of Anatomy	2.4	Q3	25	1,621	64.8
7	Biomaterials	14.0	Q1	21	1,455	69.3
8	Acta Biomaterialia	9.7	Q1	31	1,219	39.3
9	Journal of Bone and Joint Surgery. America Volume	5.3	Q1	18	1,094	60.8
10	Knee Surgery, Sports Traumatology, Arthroscopy	3.8	Q1	34	936	27.5

### 3.5 Key words

**Figure 9:**
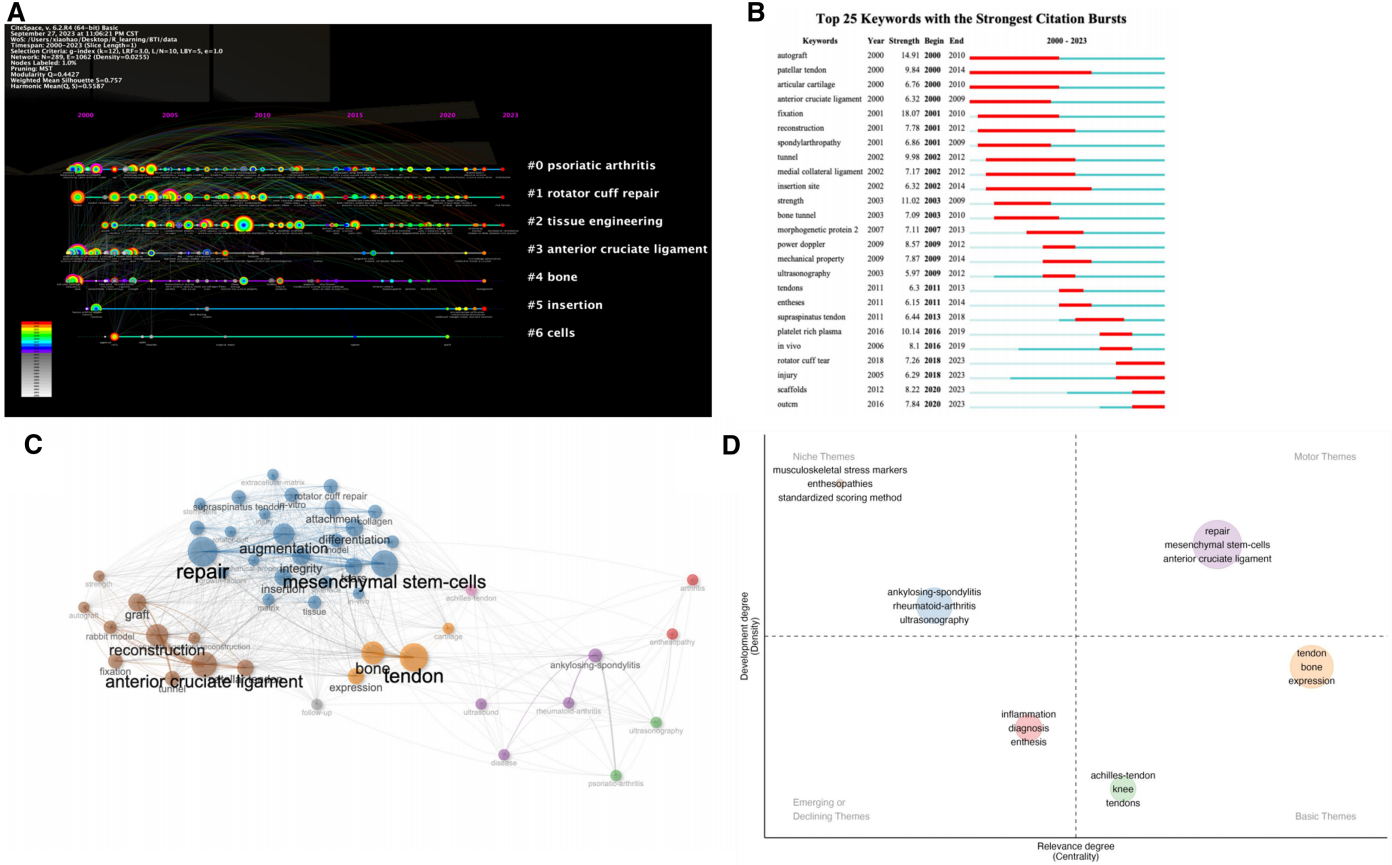
Keyword timeline graphs, citation bursts, and clustering graphs. (**A**) Keyword clustering timeline graph. (**B**) Top 25 strongest citation burst keywords. (**C**,**D**) Keyword clustering information.

Keywords can reflect the hotspots and layers in a particular field. As shown in Figure 9A, the keyword time plot analyzes a total of seven clusters, which are “psoriatic arthritis”, “rotator cuff repair”, “tissue engineering”, “anterior cruciate ligament”, “bone”, “insertion”, and “cells”. “Psoriatic arthritis”, “rotator cuff repair”, and “tissue engineering” are the most important research areas for BTI, and the key clusters included in these three fields in 2022 and 2023 are “stimulation”, “biomechanics”, “physical activities”, “gene expression”, “mechanical stimulation”, and “platelet-rich plasma”. In the analysis of the top 25 most cited outbreak keywords, we found that “rotator cuff tear”, “injury”, “scaffolds “, and “outcome” are the keywords that have occurred more frequently in the BTI field recently (Figure 9B). As shown in Figure 9, we can categorize the keywords into several major sets, respectively the “anterior cruciate ligament-reconstruction” set, the “bone-tendon-expression” set, “mesenchymal stem cells-repair” set, and “ankylosing-spondylitis” set. According to the keyword centrality and density (Figure 9D), “repair-anterior cruciate ligament-mesenchymal stem cells” has high centrality and density.

## 4 Discussion

Bibliometric analysis is an excellent methodology that provides experienced researchers with a systematic and visual knowledge structure and helps new scientific researchers to be aware of the general trends in their field of study. In this article, we have analyzed the bibliometric knowledge in the field of BTI over the last two decades.

In the time period researched, the current analysis of BTI research shows a general upward trend in the number of BTI publications. It is not difficult to infer that more and more researchers are starting to pay attention to the relationship between this particular structure and sports rehabilitation and its structurally related disorders. It can be predicted that the number of papers and the citation frequency of this field will continue to grow steadily in the next few years. Among the paper types, the number of Articles is four times higher than the number of Review Articles and Meeting Abstracts. This reflects that researchers are emphasizing more on original research rather than reviews in the field of BTI. Despite these achievements over the years, there are still limitations in understanding the specific pathogenesis and treatment of BTI-related diseases. Currently, the treatment of BTI injuries is mostly arthroscopic surgery, but it does not improve long-term treatment outcomes (32). To treat BTI injuries better, scientists are willing to spend more effort to study the intrinsic mechanisms and effective methods of restoration after BTI injuries and to search for effective therapeutic targets. Consequently, original research has accounted for approximately more than 80% of BTI-related publications in the past decade.

The total number of citations is an essential indicator of the impact of an article and the degree of attention paid to the scientific issues that are intrinsic to it. Of the 10 most influential studies, 5 were clinically relevant, and the most cited were clinical studies of BTI-related disorders. There are two possible explanations for the overwhelming focus on clinical issues: first, BTI injury and related disorders are still a serious disease, and second, basic research on BTI may have reached a bottleneck. Therefore, there is an urgent necessity for several researchers who can once again push the field forward.

The number of papers published in a particular research area is considered an important indicator for evaluating the level of scientific research in a country or organization (23). In terms of countries, the United States published the most papers, followed by the United Kingdom, China, Japan, and European countries or regions such as Germany and France. This may be related to the strong scientific research capacity of the United States and Europe as well as the large population base of China. However, when the 100 most cited papers were analyzed, there was a shift in the rankings of other countries, except the U.S. and the U.K., which maintained their first and second places. Japan and Germany were ranked third and fourth, respectively, and China was in fifth place. This is most probably due to the higher prevalence of BTI injuries and related disorders in Europe and the United States, and may also be associated with the earlier development of their related research. China ranked second in terms of the number of papers, but the average number of citations was the lowest among the top 10 countries, the H-index was also lower than the United Kingdom, which indicates that the quality of published research in China is not satisfactory. This may be attributed to the lack of research facilities in China over the past few years.

In terms of research institutions, the University of Leeds is the institution that published the most papers in the world, followed by the Hospital for Special Surgery and University of California system. MCGONAGLE D, THOMOPOULOS S, and RODEO SA have the highest number of publications and citations in the field of BTI in the world. They can be considered as leaders in the field of BTI research. Among them, THOMOPOULOS S owns the highest H-index., which indicates he's papers are high in quality. When we delve into the relationship between these three individuals, we find that they all come from different institutions, indicating that the field is not dominated by one institution. Among the top ten institutions, 90% are world-renowned universities. These results suggest that the main output of papers in the field of BTI is from universities, and several universities in the US and Europe are particularly influential in the field. Some hospitals have relatively close connections with universities, which implies that widespread partnerships between hospitals and universities can have a positive impact on the transition from research to clinical practice.

The value of transnational collaboration in promoting innovation and solving new unresolved challenges has been widely recognized globally, and transnational interactions can compare the effectiveness of different therapies and give recommendations for more effective treatment options (33, 34). The value of transnational collaboration in promoting innovation and solving new unresolved challenges has been widely recognized globally, and transnational interactions can compare the effectiveness of different therapies and give recommendations for more effective treatment options (33, 34). As mentioned above, from 2000 to 2023, many countries around the world have conducted collaborative research in the area of BTI studies. Our study indicates that the United States is the country that has the most frequent international collaborations, which highlights its influence in the field of BTI. Meanwhile, the UK has the second highest number of international collaborations after the US, indicating that the UK has a significant impact on the BTI field and is actively engaged in international collaborations in the field of BTI. The University of Leeds has extensive collaborations with the world's top research institutions in BTI research, but interestingly, the Hospital for Special Surgery, which is also in the top three in terms of the number of publications, collaborates intensively with institutions in North America. Shanghai Jiao Tong University, one of the most famous research institutes in China, is supposed to collaborate with influential research institutes around the world, but this is not the case, and its collaboration with Central South University in China is also very limited. This contradiction may be attributed to the fact that China's cooperation with other countries and regions is relatively decentralized rather than concentrated.

The most influential authors in the field of BTI research have their core research directions which represent the cutting edge and hot spots directions in the field. For example, Lu HB mainly investigated the role of mechanical stimulation and biological properties of tissue-engineered scaffolds in BTI injury restoration (35–37). The research group of Benjamin M and McGonagle D et al. primarily concentrated on clarifying the pathologic changes and their mechanisms during repair after BTI injury. They illustrated the connection between histologic changes such as inflammation and new bone formation and the formation of spondylarthritis, as well as the relationship between dermatologic and arthropathic conditions in psoriasis (38–40).

Bibliometric analysis generates journal indexes which can provide a reliable reference for researchers searching the paper and submitting manuscripts (41, 42). Our analysis shows that “The American Journal of Sports Medicine” is the most cited. “The American Journal of Sports Medicine” is one of the better publications in the field of sports medicine and is known for its depth and breadth of expertise in sports medicine. It places more emphasis on basic research in the field of BTI. “Arthroscopy-The Journal of Arthroscopic and Related Surgery” is the second most cited journal that publishes papers on BTI concentrating on clinical research on arthroscopy and its related surgeries. Thus, “Arthroscopy-The Journal of Arthroscopic and Related Surgery” is a complement to “The American Journal of Sports Medicine” in terms of the type of literature, and “The American Journal of Sports Medicine” along with “Arthroscopy-The Journal of Arthroscopic and Related Surgery” have led the way in the improvement of BTI research in this era. It is worth noting that only two of the top ten cited journals had an impact factor of more than 10, indicating that the quality of journals in the field of BTI is not satisfactory and that publishers and researchers are expected to further improve the quality of their papers. It also indicates that the BTI is not a very popular field for research, which needs more researchers’ input and discovery.

Keywords can reflect the current research topic hotspots, as well as the trend of changing research topic hotspots (43). Based on the heterogeneity among studies, we categorized the keywords into seven clusters. According to our cluster analysis, diseases involving BTI mainly include an autoimmune disease “psoriatic arthritis” “sports injuries” “rotator cuff repair” and “anterior cruciate ligament”. Injury to BTI is an important pathologic feature of psoriatic arthritis, which distinguishes psoriatic arthritis from rheumatoid arthritis (RA) (44). The restoration of damage in BTI is also a therapeutic challenge for sports injuries (4). Focusing on the key clusters appearing in 2022 and 2023, we can find several treatments that are receiving more attention from researchers involving “mechanical stimulation”, “platelet-rich plasma” and “scaffolds”. Mechanical stimulation is a type of exercise therapy that consists mainly of continuous passive movement (45), shockwave therapy (46), Low-intensity pulsed ultrasound (LIPUS) (47), pulsed electromagnetic field (PMF) (48) etc. Mechanical stimulation is an important biophysical factor in the organism and has a significant effect on the restoration of injuries at the tendon and BTI (49). During the development of the BTI, a study in birds by R. Christian Solem et al. indicated that paralysis of skeletal muscle affects the expression of fibroblast growth factor (FGF) and bone morphogenetic protein (BMP) pathways, inhibiting secondary cartilage formation at the BTI (50). After arthrotendinous interface restoration surgery, patients are also often asked to perform an adequate level of exercise to enhance their recovery (51). A randomized controlled trial demonstrated the effectiveness of Extracorporeal shock wave therapy (ESWT) in the treatment of calcific tendinitis (52). Randomized controlled trials also illustrate the repair-promoting effect of ESWT after ACL reconstruction surgery (53). Platelet-rich plasma (PRP) is widely used in BTI restoration. BTI restoration after ACL transplantation can be accelerated by utilizing bioactive platelet-rich fibrin scaffolds (54). In an animal study by Chin-Chean Wong et al, scaffolding material added PRP was observed to enhance tendon cell activity at the cellular level and to promote fibrocartilage and neoosteogenesis at the BTI (54). However, due to the limitation in the amount of PRP extracted, the application of PRP is constrained (55). Scaffolds are important supplements to grafts. Calcium or magnesium phosphate biocements or adhesives are common biomaterials that can recruit cytokines at the BTI and stimulate cell proliferation (56, 57). However, materials such as calcium phosphate take a long time to degrade in the body and may interfere with the regeneration of autologous bone cells, which can impact bone reconstruction (58). Degradable materials with porous structure can provide a surface for cell absorption, and therefore provide a suitable environment for cell growth and differentiation (59). Additionally, stent material added to PRP can promote BTI healing (45). In conclusion, more research is needed to explore treatments that accelerate BTI healing and provide better treatment options for patients with psoriatic arthritis, sports injuries, and more.

As shown in Figure 9D, mesenchymal stem cells (MSCs) are playing an increasingly important role in the treatment and reconstruction of BTI. MSCs have the ability to self-renew and differentiate into different tissues and are widely found in various tissues of the human body, where they can promote vascular regeneration, cell proliferation, and secretion of various bioactive factors. Recent studies have identified exosomes as an important mechanism of action for MSCs to promote tendon recovery (60). Interactions between MSCs and macrophages also promote rational healing of BTIs (61). Cell Sheet Technology, which preserves the intercellular junctions and extracellular matrix intact, in combination with MSCs, is not a new means of treating BTI injury (62). Therefore, future treatments regarding BTI and its rational regeneration may focus mainly on (1) Tissue engineering, (2) Regenerative medicine including MSCs, dialogue between macrophages, etc., (3) Biomechanical treatments, and (4) Emerging biotechnologies such as Cell Sheet Technology.

This study also has some shortcomings. We used only WoSCC for the bibliometric analysis, excluding other publicly and commercially available bibliometric databases such as Scopus, Medline, and PubMed, which inevitably led to the problem of incomplete data for the analysis. Due to the limitations of CiteSpace and the lack of uniform parameter setting standards, data loss and partial data overlap are inevitable during the software clustering process, which can also lead to biased analysis results. In addition, we only collected English publications, which may ignore high-quality literature in other languages. Finally, reading graphs illustrating the links between different countries and institutions is also made difficult by the close and complex cooperation between countries and institutions in this field.

## 5 Conclusion

In conclusion, there has been a general increase in the amount of published papers on BTI per year over the past two decades. The United States is the most productive country, while the University of Leeds has also achieved significant research results, which are instructive in promoting the further development of BTI. Additionally, MCGONAGLE D has the most publications in the field of BTI. Given the complexity of BTI research, the existence of close transnational relationships between countries, institutions, and authors is expected to promote innovation in research and solve problems that remain unresolved in the field. Our results provide new ideas for BTI-related research and facilitate further studies on the etiology, diagnosis, and treatment of BTI-related injuries and diseases.
